# LncRNA MILIP links YBX1 to translational activation of Snai1 and promotes metastasis in clear cell renal cell carcinoma

**DOI:** 10.1186/s13046-022-02452-9

**Published:** 2022-08-26

**Authors:** Yanliang Wang, Yu Chen Feng, Yujin Gan, Liu Teng, Li Wang, Ting La, Peilin Wang, Yue Gu, Lei Yan, Na Li, Lina Zhang, Limeng Wang, Rick F. Thorne, Xu Dong Zhang, Huixia Cao, Feng-Min Shao

**Affiliations:** 1 Department of Nephrology, Henan Provincial Key Laboratory of Kidney Disease and Immunology, Henan Provincial Clinical Research Center for Kidney Disease, Henan Provincial People’s Hospital and People’s Hospital of Zhengzhou University, Zhengzhou, China; 2 School of Medicine and Public Health, The University of Newcastle, Newcastle, Australia; 3 Translational Research Institute, Henan Provincial and Zhengzhou City Key Laboratory of Long Non-Coding RNA and Cancer Metabolism, Henan International Joint Laboratory of Non-Coding RNA and Metabolism in Cancer, Henan Provincial People’s Hospital and People’s Hospital of Zhengzhou University, Zhengzhou, China; 4 School of Basic Medicine Sciences, Academy of Medical Science, Zhengzhou University, Zhengzhou, China; 5 School of Biomedical Sciences and Pharmacy, The University of Newcastle, Newcastle, Australia

**Keywords:** Long non-coding RNA, MILIP, Clear cell renal cell carcinoma, YBX1, Snai1

## Abstract

**Background:**

Distant metastasis is the major cause of clear cell renal cell carcinoma (ccRCC)-associated mortality. However, molecular mechanisms involved in ccRCC metastasis remain to be fully understood. With the increasing appreciation of the role of long non-coding RNAs (lncRNAs) in cancer development, progression, and treatment resistance, the list of aberrantly expressed lncRNAs contributing to ccRCC pathogenesis is expanding rapidly.

**Methods:**

Bioinformatics analysis was carried out to interrogate publicly available ccRCC datasets. In situ hybridization and qRT-PCR assays were used to test lncRNA expression in human ccRCC tissues and cell lines, respectively. Chromatin immunoprecipitation and luciferase reporter assays were used to examine transcriptional regulation of gene expression. Wound healing as well as transwell migration and invasion assays were employed to monitor ccRCC cell migration and invasion in vitro. ccRCC metastasis was also examined using mouse models in vivo. RNA pulldown and RNA immunoprecipitation were performed to test RNA–protein associations, whereas RNA-RNA interactions were tested using domain-specific chromatin isolation by RNA purification.

**Results:**

MILIP expression was upregulated in metastatic compared with primary ccRCC tissues. The increased MILIP expression in metastatic ccRCC cells was driven by the transcription factor AP-2 gamma (TFAP2C). Knockdown of MILIP diminished the potential of ccRCC cell migration and invasion in vitro and reduced the formation of ccRCC metastatic lesions in vivo. The effect of MILIP on ccRCC cells was associated with alterations in the expression of epithelial-to-mesenchymal transition (EMT) hallmark genes. Mechanistically, MILIP formed an RNA-RNA duplex with the snail family transcriptional repressor 1 (Snai1) mRNA and bound to Y-box binding protein 1 (YBX1). This promoted the association between the YBX1 protein and the Snai1 mRNA, leading to increased translation of the latter. Snai1 in turn played an important role in MILIP-driven ccRCC metastasis.

**Conclusions:**

The TFAP2C-responsive lncRNA MILIP drives ccRCC metastasis. Targeting MILIP may thus represent a potential avenue for ccRCC treatment.

**Supplementary Information:**

The online version contains supplementary material available at 10.1186/s13046-022-02452-9.

## Background

Clear cell renal cell carcinoma (ccRCC) is the most common form of renal cell carcinoma and represents one of the most lethal urological malignancies [1–3]. Although partial or radical nephrectomy cures most patients with primary ccRCCs, the outcome of patients with metastatic ccRCCs remains dismal despite the recent advance in molecularly targeted therapy and immunotherapy [3–8]. Indeed, distant metastasis is the major cause of ccRCC-associated mortality [4, 9]. However, the molecular mechanisms involved in ccRCC metastasis remain to be fully understood, although it is known that the epithelial-to-mesenchymal transition (EMT) plays a central role in driving cancer metastasis, whereas snail family transcriptional repressor 1 (Snai1), a member of the Snail family of transcription factors, is key to the regulation of EMT [10–14].

Long non-coding RNAs (LncRNAs) commonly function to establish intermolecular interactions with other biomolecules, including proteins, DNAs, and other RNAs [15]. This enables lncRNAs to act as tethers, guides, decoys or scaffolds to accomplish diverse biological functions, including cancer development, progression, and treatment resistance [16–18]. For example, the lncRNA c-Myc-inducible long noncoding RNA inactivating p53 (MILIP), also known as v-maf avian musculoaponeurotic fibrosarcoma oncogene homolog G (MAFG) antisense RNA1 (MAFG-AS1), represses the tumor suppressor p53 and promotes tumorigenesis in diverse cancer types, whereas the lncRNA pan-cancer lncRNA activating NCOR2 responsive to E2F1 (PLANE), also known as melanotransferrin antisense RNA 1 (MELTF-AS1 or MFI2-AS1), regulates an alternative splicing program to promote cancer pathogenesis similarly in the pan-cancer context [19, 20]. In particular, the list of aberrantly expressed lncRNAs that contribute to ccRCC pathogenesis is also expanding rapidly [21, 22]. For instance, while the lncRNA up-regulation in clear cell renal cell carcinoma (URRCC) promotes ccRCC cell proliferation and invasion through promoting the expression of epidermal growth factor-like protein 7 (EGFL7), the lncRNA DNA methylation–deregulated and RNA m6A reader–cooperating (DMDRMR) stabilizes insulin-like growth factor 2 mRNA-binding protein 3 (IGF2BP3) target genes to facilitate ccRCC growth and metastasis [21, 22].

An emerging mode of lncRNA-mediated regulation of biological processes involves lncRNAs forming RNA-RNA duplexes to control gene expression post-transcriptionally [20, 23]. Indeed, bioinformatic prediction tools have revealed the existence of numerous duplex-forming motifs across the transcriptome, suggesting that duplex-facilitated gene regulation could be far more common than currently appreciated [24]. Here we show that the lncRNA MILIP forms an RNA-RNA duplex with the Snai1 mRNA that recruits Y-box binding protein 1 (YBX1) to facilitate YBX1-mediated translational activation of Snai1, which in turn promotes the EMT and metastasis of ccRCC cells. Moreover, we demonstrate that MILIP is frequently upregulated in metastatic ccRCCs through activating enhancer binding protein 2 (AP-2) gamma (TFAP2C; also known as AP-2γ)-mediated transcriptional activation, with practical implications of MILIP targeting for preventing ccRCC metastasis.

## Methods

### Cell culture and human tissues

The human ccRCC cell lines, 786-O, Caki-1 and ACHN were purchased from National Collection of Authenticated Cell Cultures, Chinese Academy of Sciences (Shanghai, China). The human ccRCC cell line, 769-P was purchased form iCell Bioscience lnc. (Shanghai, China). 786-O and 769-P cells were cultured in RPMI-1640 medium (Invitrogen, Cat#11875–093) supplemented with 10% fetal bovine serum (FBS, Biological Industries, Cat#04–001-1ACS; Beit Haemek, Israel) and 1% penicillin–streptomycin (Solarbio, Cat#P1400; Beijing, China). Caki-1 cells were cultured in McCoy’s 5A medium (Biological Industries, Cat#01–075-1A) with 10% FBS and 1% penicillin–streptomycin. ACHN cells were maintained in MEM (iCell Bioscience lnc, Cat#iCell-0012; Shanghai, China) with 10% FBS and 1% penicillin–streptomycin. All cells were cultured in a humidified incubator at 37 °C and 5% CO_2_. All cell lines were verified to be free of mycoplasma contamination every 3 months. Individual cell line authentication was confirmed using the AmpFISTR Identifiler PCR Amplification Kit from Applied Biosystems and GeneMarker V1.91 software (SoftGenetics LLC). The formalin-fixed paraffin-embedded (FFPE) ccRCC tissue microarray (Cat#HKid-CRC060CS-01) was purchased from the Shanghai Outdo Biotech Co., Ltd (Shanghai, China). Studies using human tissues were approved by the Human Research Ethics Committees of Henan Provincial People's Hospital (No. 2019–44) in agreement with the guidelines set forth by the Declaration of Helsinki. The study was compliant with all relevant ethical regulations for human research participants and all participants provided written informed consent.

### Antibodies and reagents

Information on antibodies used in this study is provided in Supplementary Table 1. The following reagents were purchased from indicated companies: Doxycycline (Med Chem Express, Cat#HY-N0565B; New Jersey, USA), Cycloheximide (Med Chem Express, Cat#HY-12320), Protease Inhibitor Cocktail (Cell signaling technology, Cat#5871s), RNase Inhibitor (Beyotime, Cat#R0102-10kU; Shanghai, China), 4% paraformaldehyde (Servicebio, Cat#G1101; Wuhan, China), Opti-MEM™ Reduced Serum Medium (ThermoFisher Scientific, Cat#31985070), Protease K (Solarbio, Cat#P1120; Beijing, China), Mounting medium with DAPI (Abcam, Cat#ab104139).

### Small interfering RNA and plasmid transfection

The small interfering RNAs (siRNAs) were purchased from Sangon Biotech Co., Ltd. (Shanghai, China) and transfected using Lipofectamine™ RNAiMAX transfection reagent (ThermoFisher Scientific, Cat#13778030) according to manufacturer’s instructions. Plasmids pcDNA3.1-MILIP, pcDNA3.1-MILIP-ΔDFO, pEGFP-N1-YBX1(FL), pEGFP-N1-YBX1-ΔAP, pEGFP-N1-YBX1-ΔCSD, pEGFP-N1-YBX1-ΔCST, pGL3-*MILIP* promoter and pGL3-*MILIP* promoter-ΔBR were constructed by Tsingke Biotech CO., Ltd. (Beijing, China) and transfected using Lipofectamine 3000 transfection reagent (ThermoFisher Scientific, Cat#L3000001) according to manufacturer’s instructions. siRNA sequences are listed in Supplementary Table 2.

### Inducible shRNA knockdown

Short hairpin RNA (ShRNA) oligos were constructed into the FH1-tUTG plasmid as previously described [20]. Lentivirus particles were packaged in HEK293 cells through co-transfecting FH1-tUTG inserted with shRNA oligos (6 µg), pMDLg.pRRE (6 µg, Addgene, Cat#12251), pMD2.g (6 µg, Addgene, Cat#12259) and pRSV.Rev (3 µg, Addgene, Cat#12253) plasmids. The lentiviral particles were harvested after 48 h transfection and stored in -80℃. Caki-1 or ACHN cells were transduced with the lentiviral particles in 3.5 cm cell culture dishes to establish inducible knockdown cell sublines. The knockdown of MILIP was induced in response to doxycycline treatment. ShRNA oligos were purchased from Sangon Biotech Co., Ltd. (Shanghai, China). ShRNA oligo sequences are listed in Supplementary Table 2.

### Transwell migration assay

Cells with or without MILIP knockdown were seeded to upper chambers of 24-well transwell inserts (8 µm; Corning, Cat#34212; 5 × 10^4^ cells/well) and maintained in serum-free medium. The bottom chambers were filled with medium supplemented with 10% FBS. After 48 h, cells grown in inserts were fixed with 4% paraformaldehyde (Servicebio, Cat#G1101; Wuhan, China) for 10 min and then stained using 1% crystal violet stain solution (Solarbio, Cat#G1062; Wuhan, China) for 15 min. The non-migrated cells on the upper surface of the membranes were removed by scrubbing with a cotton tipped swab. Cells that migrated to the lower surface of the membranes were further imaged using Macro Zoom Fluorescence Microscope System (Olympus, MVX10) prior to quantification with the ImageJ software.

### Transwell invasion assay

Cell invasion was assayed using inserts pre-coated with 7% Matrigel (BD Biosciences, Cat#BD-356234). Cells with or without MILIP knockdown were loaded in the upper chambers of 24-well transwell inserts (1 × 10^5^ cells/well) and maintained in serum-free medium. The bottom chambers were filled with medium supplemented with 10% FBS. After 24 h, cells were fixed and stained as described in the migration assay. After removing the non-invaded cells on the upper surface of the membranes, the invasive cells were imaged and quantified following the methods in the migration assay.

### Wound healing assay

Cells with or without MILIP knockdown were grown to 90% confluence, and then monolayers were scratched with 200 µl pipette tips across the center of wells. After being washed with PBS for three times, cells were then maintained in the serum-free medium and imaged over a 24 h period at 12 h intervals to monitor wound width. The wound width was quantified using the Vision Works software (Analytik Jena, Jena, Germany).

### RNA extraction and quantitative real-time PCR (qRT-PCR)

Total RNA from ccRCC cells was extracted with Trizol reagent (Solarbio, Cat#R1100; Beijing, China) according to manufacturer’s instructions. cDNA was then synthesized from 1 µg of total RNA using the HiScript II 1^st^ Strand cDNA Synthesis Kit (Vazyme, Cat#R211; Nanjing, China). Of the resultant cDNA, 25 ng was used for qRT-PCR. The qRT-PCR mix contains 10 μl RealStar Green Power Mixture (GenStar, Cat#A313; Beijing, China), 0.25 μM of each primer, and cDNA with a total volume of 20 μl. Samples were amplified for 40 cycles using the Applied Biosystems® 7500 Real-Time PCR System (ThermoFisher Scientific). 2^−ΔΔCT^ method was used to calculate the relative gene expression levels normalized against the housekeeping gene β-Actin. Primer sequences are listed in Supplementary Table 3.

### Western blotting

Cells were washed with PBS twice and lysed in RIPA lysis buffer (Beyotime, Cat#P0013B; Shanghai, China) for 15 min on ice followed by centrifugation at 12,000 rpm for 15 min at 4 °C. Protein concentrations were measured using the BCA Protein Assay Kit (Solarbio, Cat #PC0020; Beijing, China) following the manufacturer's instructions. 20 μg proteins were resolved by 10% SDS-PAGE and transferred onto PVDF membranes (Millipore, Cat# IPVH00010). Membranes were blocked with 5% bovine serum albumin (BSA) for 1 h at room temperature (RT) and incubated with primary antibodies overnight at 4 °C followed by incubation with HRP-labeled secondary antibodies for 1 h at RT. BeyoECL Star kit (Beyotime, Cat#P0018AS; Shanghai, China) was used for protein detection. Information of antibodies used in this study is shown in Supplementary Table 1.

### Biotin RNA pulldown assay

The RNA pulldown assay was performed as previously described [25]. In brief, Caki-1 and ACHN cell pellets were lysed in lysis buffer [50 mM Tris–HCl (PH 7.5), 150 mM NaCl, 2.5 mM MgCl_2_, 1 mM EDTA, 10% Glyceral, 0.5% Nonidet P-40/Igepal CA-630, 1 mM DTT, RNase Inhibitor and Protease Inhibitor], followed by sonication for 3 cycles with 10 s on/30 s off. Cell lysates were then rotated with streptavidin beads (ThermoFisher Scientific, Cat#20349) coated with 4 μg antisense/scramble biotin-labeled probes for 4 h at 4 °C. Beads were then washed with lysis buffer for 6 times followed by RNA isolation and immunoblotting analysis. Information of antibodies and probes used in this study is shown in Supplementary Table 1 and Supplementary Table 4, respectively.

### RNA immunoprecipitation (RIP) assay

The RIP assay was performed using the Magna RIP™ RNA-Binding Protein Immunoprecipitation Kit (Millipore, Cat#17–701) following instructions provided by the manufacturer. In brief, 2 × 10^7^ cells were lysed with equal pellet volume of RIP lysis buffer. 100 μl cell lysate was incubated with Pierce™ Protein A/G Agarose (ThermoFisher Scientific, Cat#20422) pre-coated with YBX1 antibodies at 4 °C overnight. Normal rabbit or mouse IgG was used as the negative control. For cells transfected with pEGFP-N1-YBX1(FL), pEGFP-N1-YBX1-ΔAP, pEGFP-N1-YBX1-ΔCSD or pEGFP-N1-YBX1-ΔCST plasmids, the lysates were incubated with anti-GFP mAb-Magnetic Agarose (MBL, Cat#D153-10; Nagoya, Japan). After being washed with RIP wash buffer, beads-bound immunocomplexes were prior to RNA isolation and immunoblotting analysis. Information of antibodies and primers used in this study is shown in Supplementary Table 1 and Supplementary Tables 3&4, respectively.

### Domain-specific chromatin isolation by RNA purification (dChIRP)

dChIRP assays were performed as previously described [20]. Briefly, Caki-1 and ACHN cells were harvested and cross-linked in 1% glutaraldehyde for 10 min at RT. Cell pellets were then lysed in lysis buffer (50 mM Tris–HCl [pH 7.0], 10 mM EDTA, 1% SDS, PMSF, Superase-in) on ice for 30 min, prior to sonication for three cycles (10 s on/30 s off). 4 µg antisense/scramble biotin-labelled probes against MILIP RNA was rotated with cell lysates at 37 °C for 4 h, followed by further incubating with 100 µl C-1 magnetic beads (Invitrogen, Cat #65002) at 37 °C for 30 min. Beads were then washed in wash buffer for five times, followed by RNA isolation. For cell free system, dChIRP assays were performed using 1 µg in vitro-transcribed biotin-labeled MILIP RNA or MILIP RNA with its DFO deleted incubated with 1 µg Snai1 RNA or Snai1 RNA with MILIP-BR deleted. In vitro-transcribed MILIP RNA without biotin labeling was included as a negative control. Primer and probe sequences are shown in Supplementary Table 3 and Supplementary Table 4, respectively.

### In situ hybridization (ISH)

The ISH assays were performed with the RNAscope® 2.5 HD Detection Reagent-Brown kit (Advanced cell diagnostics, Cat#322310) according to manufacturer’s instructions. In brief, the FFPE ccRCC tissue microarray was deparaffinized, rehydrated, boiled in target retrieval solution, and treated with proteinase K. Sections were then incubated with probes (Advanced Cell Diagnostics, Cat#547581) for 4 h at 40 °C. After being washed, sections were incubated with 3,3’-diaminobenzidine (DAB), and counterstaining was performed using hematoxylin. Quantification was performed by examining the percentage of positive cells ranged from 0 to 100%. The intensity of staining (intensity score) was judged on an arbitrary scale of 0–4: no staining (0), weakly positive staining (1), moderately positive staining (2), strongly positive staining (3) and very strong positive staining (4). A reactive score (RS) was derived by multiplying the percentage of positive cells with staining intensity divided by 10.

### Subcellular fractionation

Subcellular fractionation was performed as previously described [19, 20]. 1 × 10^7^ cells were harvested and lysed in 600 μl hypotonic buffer I (25 mM Tris–HCl, PH 7.4, 1 mM MgCl_2_, 5 mM KCl) for 5 min on ice. 600 μl hypotonic buffer II (25 mM Tris–HCl, PH 7.4, 1 mM MgCl_2_, 5 mM KCl, 1% NP-40) was then added to lysates and incubated for further 5 min on ice. After centrifugation at 5,000 × g for 15 min, supernatants were collected as cytoplasmic fractions. The pellets were washed with cold PBS followed by nuclear fractionation using nucleus resuspension buffer (20 mM HEPES, PH 7.9, 400 mM NaCl, 1 mM EDTA, 1 mM EGTA, 1 mM DTT, 1 mM PMSF). Supernatants were harvested as nuclear fractions after centrifugation at 12,000 × g for 10 min.

### In vitro transcription

The in vitro transcription assay was performed as previously described [20]. DNA templates for in vitro transcription were generated by PCR amplification using forward primers containing the T7 RNA polymerase promoter sequence and reverse primers without the promoter sequence. In vitro transcription was then performed using the TranscriptAid T7 High Yield Transcription Kit (Thermofisher Scientific, Cat#K0441) following the manufacturer’s instructions. Primer sequences are listed in Supplementary Table 5.

### Chromatin immunoprecipitation assay (ChIP)

The ChIP assay was performed using the Chromatin Immunoprecipitation (ChIP) Assay Kit (Beyotime, Cat#P2078; Shanghai, China) according to manufacturer’s instructions. In brief, cells crosslinked with 1% formaldehyde for 10 min at 37 °C were lysed and sonicated. Cell lysates were then rotated with an anti-TFAP2C antibody or a corresponding anti-normal rabbit IgG antibody at 4 °C overnight. Then, 60 μl of protein A/G agarose beads was added to the antibody-lysate mixture and rotated for 1 h at 4 °C. Beads were washed, and DNA fragments were eluted, purified and analyzed by RT-PCR with specific primers. Information of antibodies and primers used in this study is shown in Supplementary Table 1 and Supplementary Table 4, respectively.

### Luciferase reporter assay

Luciferase reporter assays were performed using the Dual-Luciferase Reporter Assay System (Promega, Cat#E1910) according to the manufacturer’s instruction. In brief, cells were co-transfected with pGL3-*MILIP* promoter reporters or pGL3-*MILIP* promoter-ΔBR reporters, together with pGL4.73[hRluc/SV40] reporters expressing the renilla luciferase. Firefly and renilla luciferase activities were examined after 24 h transfection.

### Mouse models of metastasis

1.5 × 10^6^ ACHN.shMILIP.1 cells were injected via the tail vein into 5-week-old male NOD/SCID mice (Nanjing GemPharmatech Co., Ltd Company, China) (eight mice per group). Drinking water containing doxycycline (1 mg/ml, supplemented with 10 mg/ml sucrose) was used to induce MILIP knockdown, whereas drinking water containing PBS was included as the control. After 7 weeks of transplantation, mice were sacrificed, and lung tissues were excised. Studies on animals were conducted in accordance with relevant guidelines and regulations and were approved by the Animal Research Ethics Committee of Zhengzhou University (Zhengzhou, China). All mice were housed in a temperature-controlled room (21–23 °C) with 40–60% humidity and a light/dark cycle of 12 h/12 h.

### Statistical Analysis

All statistical analysis was performed using Graphpad Prism 8 and Microsoft Excel software to assess differences among experimental groups. Statistical differences were analyzed by two-tailed Student’s *t*-test or one-way ANOVA test followed by Tukey’s multiple comparisons. *P* values < 0.05 were considered to be statistically significant.

## Results

### TFAP2C drives MILIP expression that is upregulated in metastatic ccRCCs

**Fig. 1:**
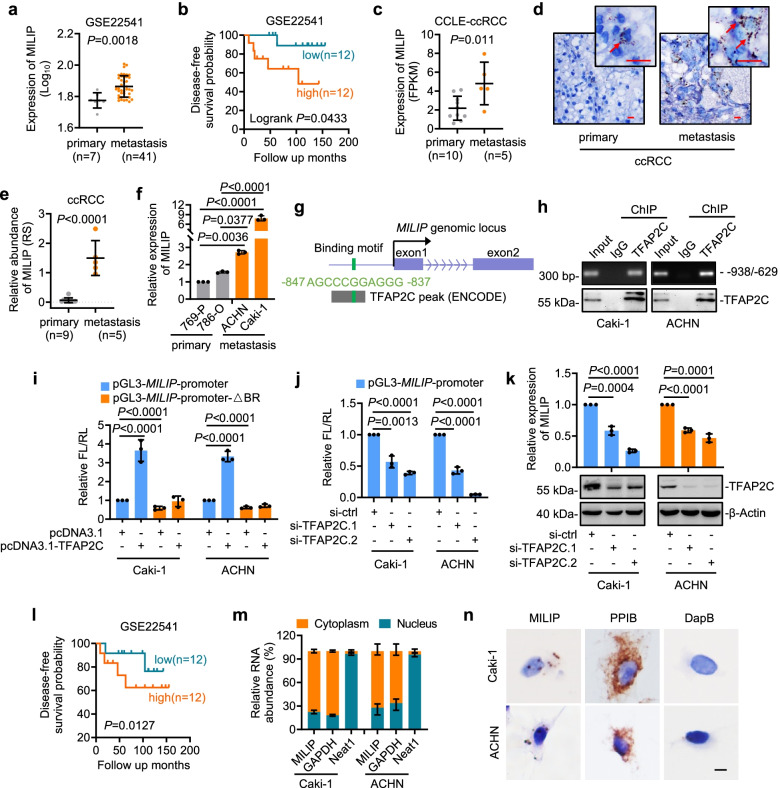
MILIP is expressed at higher levels in metastatic ccRCC and is transcriptionally activated by TFAP2C **a, b** MILIP was expressed at higher levels in metastatic compared with primary ccRCC tissues and its high level was associated with poor patient prognosis as revealed by analysis of the transcriptome array data from a GEO dataset (GSE22541). Two-tailed Student’s *t*-test (**a**). The log rank test (**b**). **c** Analysis of RNA-seq data of Cancer Cell Line Encyclopedia (CCLE) dataset showing that MILIP was expressed at higher levels in metastatic compared with primary ccRCC cell lines. Two-tailed Student’s *t*-test. **d, e** Representative microphotographs (**d**) and quantitation (**e**) of in situ hybridization (ISH) analysis of MILIP expression in metastatic compared with primary ccRCC tissues. The red arrows indicate MILIP. Scale bar, 20 µm. RS: reactive score. Two-tailed Student’s *t*-test. **f** MILIP was expressed at higher levels in metastatic than primary ccRCC cell lines. One-way ANOVA followed by Tukey’s multiple comparisons test. **g** A schematic illustration of a consensus TFAP2C binding region (TFAP2C-BR) and its ChIP-seq peak in *MILIP* promoter. **h** ChIP-PCR analysis of the association between endogenous TFAP2C and the TFAP2C-BR. **i, j** Overexpression of TFAP2C increased whereas knockdown of TFAP2C decreased the transcriptional activity of a *MILIP* reporter construct with the intact TFAP2C-BR (pGL3-*MILIP*-promoter) but not with the TFAP2C-BR deleted (pGL3-*MILIP*-promoter-ΔBR). FL: firefly luciferase activity; RL: renilla luciferase activity. One-way ANOVA followed by Tukey’s multiple comparisons test. **k** Silencing TFAP2C downregulated MILIP expression. One-way ANOVA followed by Tukey’s multiple comparisons test. **l** Kaplan-Meier analysis showing that high TFAP2C level was associated with poor patient prognosis using median of TFAP2C level as the cutoff. The log rank test. **m, n** qPCR (**m**) and ISH (**n**) analysis showing that MILIP was predominantly localized in the cytoplasm. GAPDH: cytoplasmic marker; Neat1: nuclear marker; DapB: negative control; PPIB: positive control. Scale bar, 10 µm. Data are representatives or mean ± s.d.; n = 3 independent experiments

Through interrogating the ccRCC dataset (GSE22541) included in the Gene Expression Omnibus (GEO) repository, we found that the lncRNA MILIP, which is upregulated and drives tumorigenesis in diverse cancer types, was expressed at higher levels in ccRCC tissues derived from metastatic lesions compared with those from primary tumors (Fig. 1a) [19, 26]. Moreover, high MILIP expression, similar to the occurrence of metastasis, was associated with poor disease-free survival (DFS) of ccRCC patients (Fig. 1b). Analysis of the ccRCC cell line dataset of the Cancer Cell Line Encyclopedia (CCLE) revealed that MILIP was expressed at higher levels in ccRCC cell lines generated from metastatic tumors compared with those from primary tumors (Fig. 1c). We confirmed that MILIP expression was increased in metastatic compared with primary ccRCCs using ISH in a cohort of FFPE ccRCC samples (Fig. 1d, e, Supplementary Table 6). Similarly, MILIP expression levels were higher in cell lines derived from metastatic (Caki-1 and ACHN) compared with those from primary ccRCCs (769-P and 786-O) as shown by qPCR analysis (Fig. 1f).

MILIP is transcriptionally activated by c-Myc in some other types of cancer cells [19]. However, knockdown of c-Myc did not impinge on the expression of MILIP in Caki-1 and ACHN cells (Supplementary Fig. 1a), suggesting that c-Myc does not have a major role in regulating MILIP expression in ccRCC cells. In accordance, MILIP expression levels were not correlated with c-Myc gene expression in the GSE22541 dataset (Supplementary Fig. 1b). To gain insights into transcriptional mechanisms involved in the regulation of MILIP expression in ccRCC cells, we interrogated its promoter for transcription factor binding sites using bioinformatics [19]. This revealed a consensus binding motif for the transcription factor TFAP2C located to the -837/-847 region of the promoter of the *MILIP* gene (Fig. 1g). Analysis of chromatin immunoprecipitation sequencing (ChIP-seq) data from the Encyclopedia of DNA Elements (ENCODE) Consortium revealed a TFAP2C binding peak at the TFAP2C-binding region (TFAP2C-BR) (Fig. 1g). Indeed, this region was co-precipitated with endogenous TFAP2C and was required for transcriptional upregulation of MILIP as the transcriptional activity of a *MILIP* reporter construct was inhibited when the TFAP2C-BR was deleted in Caki-1 and ACHN cells (Fig. 1h, i). Moreover, knockdown of TFAP2C diminished transcriptional activity of *MILIP* reporter in Caki-1 and ACHN cells (Fig. 1j), supporting the notion that MILIP is transcriptionally activated by TFAP2C through the identified TFAP2C-BR. Substantiating this, knockdown of TFAP2C reduced the endogenous MILIP levels (Fig. 1k, Supplementary Fig. 1c). In addition, like the high expression of MILIP, high TFAP2C expression was associated with poor DFS of ccRCC patients in the GSE22541 dataset (Fig. 1l).

Of the two annotated isoforms of MILIP (Supplementary Fig. 1d), the longer isoform (MILIP-001) was readily detected, whereas the shorter isoform (MILIP-002) was barely measurable with exon-specific primers in ccRCC cells (Supplementary Fig. 1d, e), consistent with the notion that the longer isoform is the major functional variant of MILIP [19]. Similar to previous results in other types of cancer cells, MILIP was predominantly located to the cytoplasm in ccRCC cells as shown by qPCR analysis of subcellular fractions and in situ hybridization (ISH) analysis of Caki-1 and ACHN cells grown on coverslips (Fig. 1m, n) [19]. Noticeably, although the gene encoding MILIP is located head-to-head with the *MAFG* gene on chromosome 17 separated by a short distance of 117 bp, neither knockdown nor overexpression of MILIP affected MAFG expression levels (Supplementary Fig. 1f-i) [19]. Furthermore, MILIP expression levels were not correlated with MAFG mRNA expression levels in the GSE22541 dataset (Supplementary Fig. 1j), indicating that there is no regulatory relationship between MILIP and its neighboring gene *MAFG* in ccRCC cells.

### MILIP promotes ccRCC metastasis

**Fig. 2:**
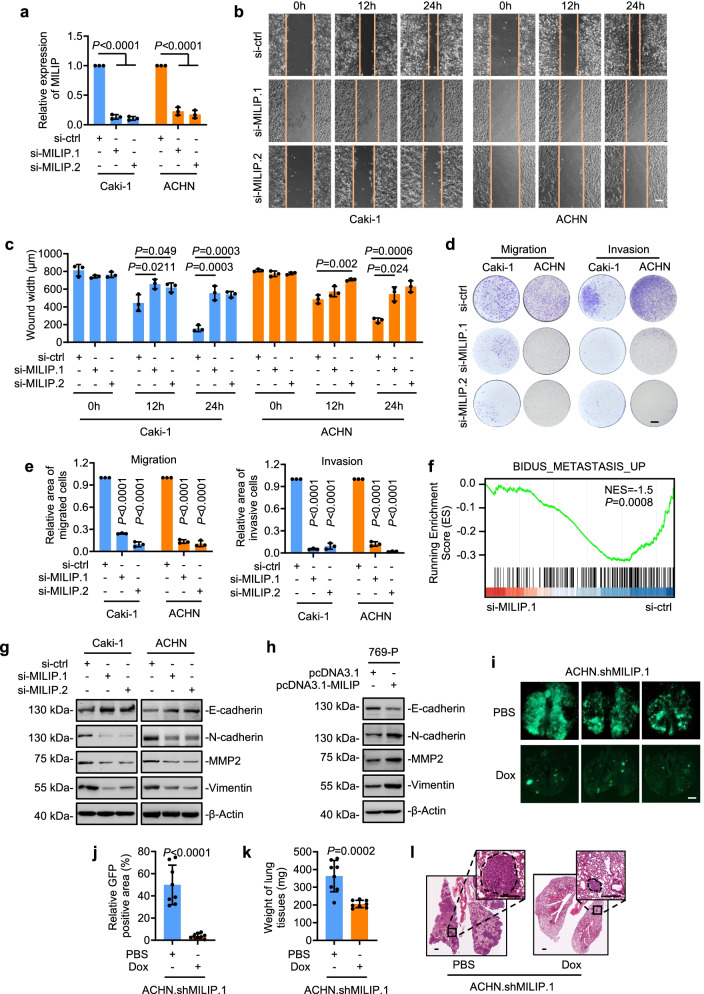
MILIP promotes ccRCC metastasis **a-e** SiRNA knockdown of MILIP (**a**) inhibited wound healing (**b, c**) and migration and invasion (**d, e**) of Caki-1 and ACHN cells. Wound width and area of migrated and invasive cells were quantitated using the ImageJ software. Scale bars, 200 μm (wound healing assay) and 1 mm (cell migration and invasion assay). One-way ANOVA followed by Tukey’s multiple comparisons test. **f** Gene Set Enrichment Analysis (GSEA) of RNA-seq data from Caki-1 cells with or without MILIP knockdown showing that the metastasis pathway was significantly downregulated after MILIP knockdown. n = 3 experimental repeats. NES, normalized enrichment score. **g** SiRNA knockdown of MILIP increased E-cadherin levels whereas decreased N-cadherin, MMP2 and Vimentin levels in Caki-1 and ACHN cells. **h** Overexpression of MILIP caused the decrease of E-cadherin levels and the increase of N-cadherin, MMP2 and Vimentin levels in 769-P cells. **i, j** Representative green fluorescence (GFP) photographs (**i**) of lung tissues from NOD/SCID mice injected with ACHN.shMILIP.1 cells via the tail vein. Mice in different groups were treated with doxycycline (Dox, 2 mg/ml supplemented with 10 mg/ml sucrose in drinking water) and PBS, respectively. GFP positive area and whole lung area were quantitated using the ImageJ software (**j**). Scale bar, 2 mm. n = 8 biologically independent animals; Two-tailed Student’s *t*-test. **k** Dox treatment reduced weights of lung tissues from NOD/SCID mice injected with ACHN.shMILIP.1 cells via the tail vein. n = 8; Two-tailed Student’s *t*-test. **l** Representative photographs of Hematoxylin and Eosin (H&E) staining of lung tissues from NOD/SCID mice injected with ACHN.shMILIP.1 cells. Scale bar, 0.5 mm. Data are representatives of eight biologically independent animals. Data are representatives or mean ± s.d.; n = 3 independent experiments or 8 biologically independent animals

Having found that MILIP is specifically upregulated in metastatic ccRCCs, we focused on investigation of the potential effect of MILIP on ccRCC cell invasion and metastasis. SiRNA knockdown of MILIP markedly delayed wound healing of Caki-1 and ACHN cells in scratch assays and reduced their migration and invasion into Matrigel in transwell assays (Fig. 2a-e). In contrast, overexpression of MILIP promoted 769-P cell wound healing, migration and invasion (Supplementary Fig. 2a-e). Indeed, gene set enrichment analysis (GSEA) of the RNA-sequencing (RNA-seq) data from Caki-1 cells revealed that MILIP knockdown caused reductions in the metastasis pathway gene signature (Fig. 2f, Additional file 1). Moreover, knockdown of MILIP led to upregulation of the epithelial marker E-cadherin and downregulation of the mesenchymal makers N-cadherin, matrix metalloproteinase-2 (MMP2) and Vimentin in Caki-1 and ACHN cells, whereas overexpression of MILIP resulted in reduction in E-cadherin and concurrent upregulation of N-cadherin, MMP2 and Vimentin expression in 769-P cells (Fig. 2g, h, Supplementary Fig. 2f, g). Together, these results suggest that MILIP promotes cell invasion and migration, which is associated with enhanced EMT, in ccRCC cells.

To facilitate further investigations, we established Caki-1 and ACHN sublines (Caki-1.shMILIP and ACHN.shMILIP) with conditional knockdown of MILIP in response to doxycycline (Dox) (Supplementary Fig. 2 h). Induced knockdown of MILIP similarly triggered reductions in cell migration and invasion in Caki-1.shMILIP and ACHN.shMILIP cells (Supplementary Fig. 2i, j). Moreover, Dox treatment of NOD/SCID mice that received tail vein injection of ACHN.shMILIP cells markedly reduced the formation of pulmonary metastatic lesions (Fig. 2i-l), demonstrating that MILIP promotes ccRCC metastasis in vivo, consistent with its role in regulating the EMT in ccRCC cells identified in vitro (Fig. 2g, h, Supplementary Fig. 2f, g).

### MILIP interacts with Y-Box Binding Protein 1 (YBX1) and promotes translational activation of Snai1

**Fig. 3:**
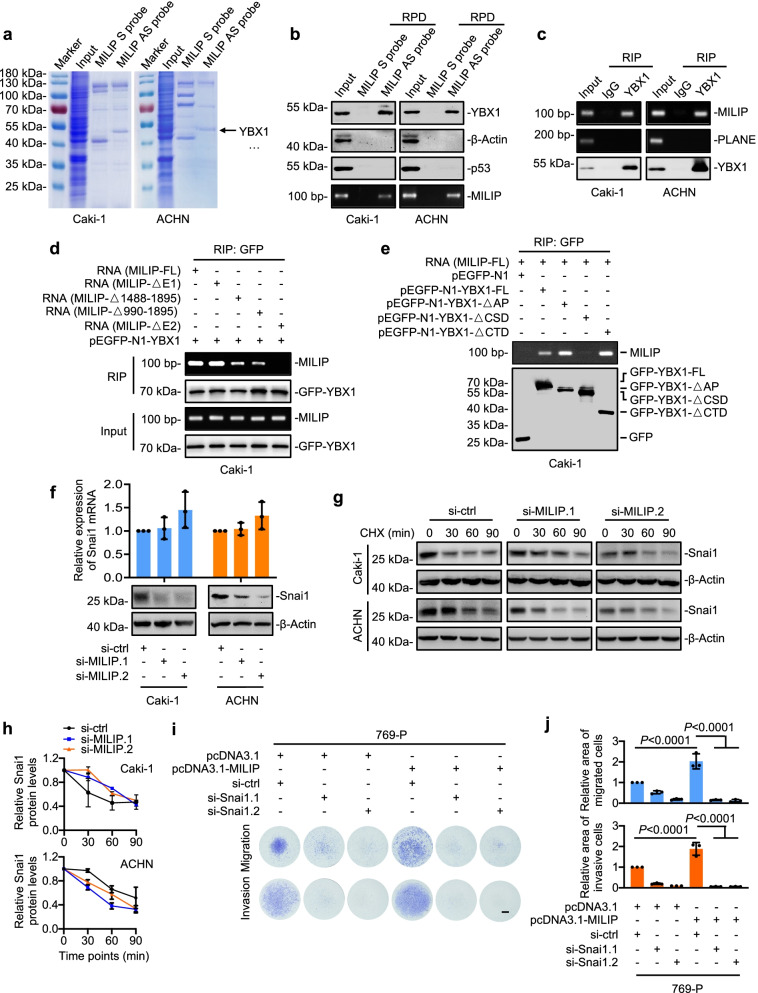
MILIP interacts with YBX1 and promotes translational activation of Snai1 **a** RNA pulldown followed by mass spectrometry analysis identified that YBX1 was the most abundant protein co-pulled down with MILIP antisense probes. S: scramble; AS: antisense. n = 1. **b** YBX1, but not p53, was co-pulled down with MILIP as shown in RNA pull-down (RPD) assays. β-Actin was included as a negative control. **c** MILIP was co-precipitated with YBX1 as detected by RNA immunoprecipitation (RIP) assays. LncRNA PLANE was used as a negative control. **d** In vitro-transcribed full-length (FL) MILIP and MILIP deletion mutants MILIP-ΔE1, MILIP-Δ1488-1895, MILIP-Δ990-1895 but not MILIP-ΔE2 were co-precipitated with GFP-YBX1 as shown in RIP assays. MILIP-ΔE1: a MILIP mutant with its exon1 deleted; MILIP-ΔE2: a MILIP mutant with its exon2 deleted. **e** MILIP was co-precipitated with full-length (FL) YBX1, YBX1 Δ Ala/Pro-rich N-terminal domain (AP) and YBX1 Δ C-terminal domain (CTD) but not YBX1 Δ cold shock domain (CSD) as shown in RIP assays. **f** SiRNA knockdown of MILIP reduced Snai1 protein levels but not its mRNA levels. One-way ANOVA followed by Tukey’s multiple comparisons test. **g, h** SiRNA knockdown of MILIP did not impinge on the half-life of Snai1 protein (**g**). Relative abundance of Snai1 protein levels normalized to respective β-Actin levels were quantitated using the ImageJ software (**h**). CHX, cycloheximide: 20 μg/ml. One-way ANOVA followed by Tukey’s multiple comparisons test. **i, j** The increased cell migration and invasion caused by MILIP overexpression was diminished by co-knockdown of Snai1 (**i**). Relative area of migrated and invasive cells was quantitated using the ImageJ software (**j**). One-way ANOVA followed by Tukey’s multiple comparisons test. Data are representatives or mean ± s.d.; n = 3 independent experiments

To understand the mechanisms responsible for MILIP-mediated promotion of the EMT in ccRCC cells, we interrogated proteins that interact with MILIP using RNA pulldown followed by mass spectrometry analysis. The most abundant protein that coprecipitated with MILIP was YBX1 (Fig. 3a, Supplementary Tables 7 & 8), a nucleic acid-binding protein known to activate Snai1 translationally that downregulates E-cadherin expression through transcriptional repression and thus promotes the EMT [13]. The association between MILIP and YBX1 was readily confirmed using RNA pulldown and RNA immunoprecipitation (RIP) assays in Caki-1 and ACHN cells (Fig. 3b, c). In contrast, no association was detected between MILIP and β-Actin that was included as a control (Fig. 3b). Similarly, there was no association between YBX1 and the nuclear lncRNA PLANE used as an additional control (Fig. 3c) [20]. Of note, MILIP did not bind to p53 in ccRCC cells as observed in other cell types (Fig. 3b), consistent with the notion that lncRNAs function in a highly cell type-specific manner [19, 27].

To define the region of MILIP responsible for its interaction with YBX1, we carried out mapping experiments with MILIP mutants transcribed in vitro. This analysis showed that MILIP fragment 618–989 within its exon 2 (E2) was critical for its binding to YBX1 (Fig. 3d, Supplementary Fig. 3a). On the other hand, deletion of the cold shock domain (CSD) of YBX1 diminished its association with MILIP (Fig. 3e, Supplementary Fig. 3b), in agreement with the notion that the CSD is responsible for binding of YBX1 to nucleic acid [28].

Of note, knockdown of MILIP caused downregulation of Snai1 protein levels without impinging on Snai1 mRNA expression (Fig. 3f), recapitulating the effect of knockdown of YBX1 (Supplementary Fig. 4a). Cycloheximide (CHX)-chase experiments showed that knockdown of MILIP did not alter the turnover rate of the Snai1 protein (Fig. 3g, h), indicating that MILIP, similar to YBX1, functions to promote translational activation of Snai1 [13]. Indeed, knockdown of Snai1 abolished MILIP overexpression-induced ccRCC cell migration and invasion (Fig. 3i, j), suggesting that Snai1 is critical for MILIP-mediated promotion of ccRCC cell metastasis.

### MILIP forms an RNA-RNA duplex with Snai1 mRNA

**Fig. 4:**
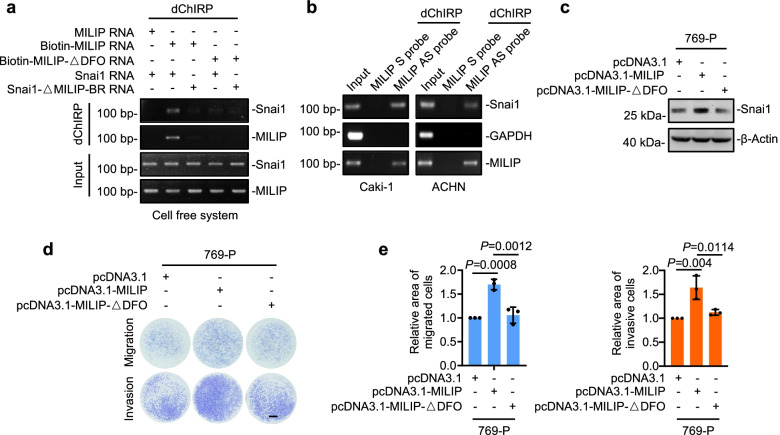
MILIP forms an RNA-RNA duplex with Snai1 mRNA to promote Snai1 translation **a** In vitro-synthesized MILIP bound to in vitro-transcribed Snai1 mRNA as shown in domain-specific chromatin isolation by RNA purification (dChIRP) assays. This binding was abolished when the MILIP-BR at Snai1 mRNA or the DFO within MILIP were deleted (Snai1-ΔMILIP-BR and MILIP-ΔDFO, respectively). BR, binding region. DFO, duplex-forming oligonucleotides. **b** Endogenous MILIP co-precipitated the endogenous Snai1 mRNA in Caki-1 and ACHN cells as shown in dChIRP assays. GAPDH protein was included as a negative control. S: scramble; AS: antisense. **c-e** Overexpression of wild-type MILIP but not MILIP with DFO deleted increased Snai1 protein expression levels (**c**) and cell migration and invasion (**d, e**) in 769-P cells. Relative area of migrated and invasive cells was quantitated using the ImageJ software. Scale bar, 1 mm. One-way ANOVA followed by Tukey’s multiple comparisons test. Data are representatives or mean ± s.d.; n = 3 independent experiments

We examined how MILIP functions to promote translational activation of Snai1. Bioinformatics analysis using the IntaRNA program (http://rna.informatik.uni-freiburg.de) identified a potential MILIP-binding region (MILIP-BR) on the Snai1 mRNA near its 5′ untranslated region (5’UTR) that complements to a fragment enriched of duplex-forming oligonucleotides (DFO) contained in MILIP (Supplementary Fig. 5a). Indeed, in vitro-synthesized biotin-labeled MILIP RNA precipitated an RNA fragment containing the MILIP-BR, but not an RNA fragment that did not have the MILIP-BR in a cell-free system (Fig. 4a). However, this association was diminished when the fragment enriched of DFO within MILIP was deleted (Fig. 4a). Therefore, MILIP and the Snai1 mRNA form an RNA-RNA duplex through the DFO and the MILIP-BR, respectively. In support, endogenous MILIP precipitated the Snai1 mRNA from Caki-1 and ACHN cell extracts (Fig. 4b).

We also tested the functional importance of the RNA-RNA duplex in MILIP-mediated regulation of Snai1 translation. In contrast to overexpression of wild-type MILIP (Fig. 4c), introduction of a MILIP mutant lacking the DFO (MILIP-ΔDFO) into 769-P cells did not impinge on Snai1 protein expression levels and did not affect cell migration and invasion as did the introduction of wild-type MILIP (Fig. 4c-e). Thus, the formation of the RNA-RNA duplex is required for MILIP-mediated promotion of Snai1 translation.

### MILIP links YBX1 to translational activation of Snai1

**Fig. 5:**
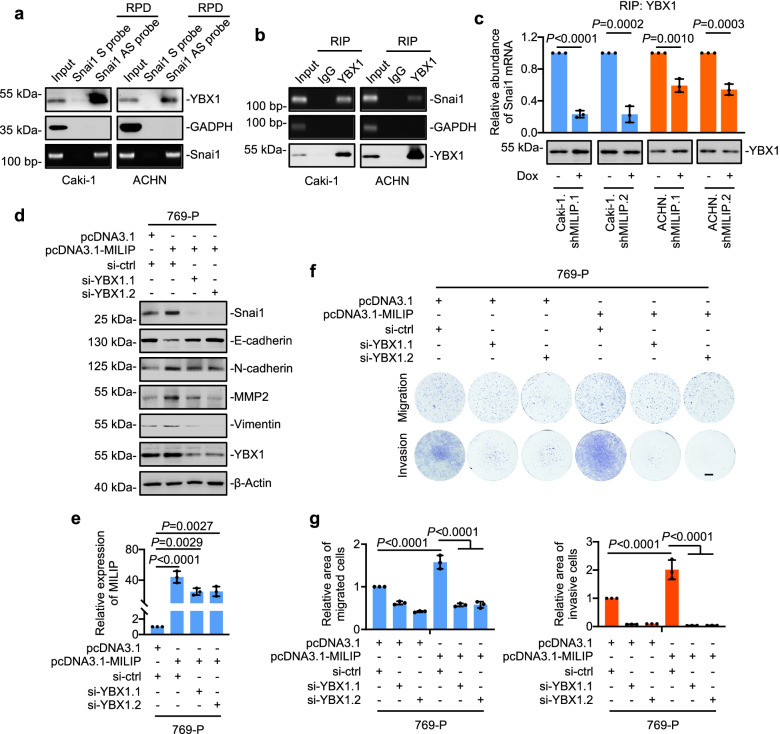
MILIP links YBX1 to translational activation of Snai1 **a** YBX1 was co-pulled down with Snai1 mRNA in Caki-1 and ACHN cells as shown in RNA pulldown (RPD) assays. GAPDH was included as a negative control. S: scramble; AS: antisense. **b** Snai1 mRNA was co-precipitated with YBX1 as detected by RNA immunoprecipitation (RIP) assays. GAPDH protein was used as a negative control. **c** Induced knockdown of MILIP decreased the amount of Snai1 mRNA associated with YBX1 protein as shown in RIP assays. Two-tailed Student’s *t*-test. **d, e** SiRNA knockdown of YBX1 diminished the increase of Snai1, N-cadherin, MMP2 and Vimentin and the decrease of E-cadherin caused by MILIP overexpression in 769-P cells. **f, g** SiRNA knockdown of YBX1 diminished the increase of cell migration and invasion caused by MILIP overexpression in 769-P cells. Relative area of migrated and invasive cells was quantitated using the ImageJ software (**g**). One-way ANOVA followed by Tukey’s multiple comparisons test. Data are representatives or mean ± s.d.; n = 3 independent experiments

We next investigated the relationship between MILIP and YBX1 in regulating translation of the Snai1 mRNA. As anticipated, YBX1 bound to the Snai1 mRNA as shown in RNA pulldown and RIP assays in Caki-1 and ACHN cells (Fig. 5a, b) [13]. Nevertheless, this association between YBX1 and the Snai1 mRNA was diminished in cells with MILIP knockdown (Fig. 5c), suggesting that MILIP facilitates the association of YBX1 with the Snai1 mRNA. Consistently, knockdown of YBX1 abolished MILIP overexpression-triggered upregulation of Snai1, N-cadherin, MMP2 and Vimentin and downregulation of E-cadherin in 769-P cells (Fig. 5d, e, Supplementary Fig. 5b). Moreover, knockdown of YBX1 blocked, at least in part, the MILIP-mediated increase in migration and invasion in 769-P cells (Fig. 5f, g). Of note, overexpression of MILIP-ΔDFO did not impinge on the expression levels of Snai1, N-cadherin, MMP2, Vimentin and E-cadherin proteins, nor did it affect cell migration and invasion in 769-P cells with or without YBX1 knockdown (Supplementary Fig. 5c-g). Together, these results demonstrate that MILIP facilitates the binding of YBX1 with Snai1 mRNA and promotes YBX1-mediated translational activation of Snai1.

## Discussion

Early metastasis is the leading cause of ccRCC-associated death, whereas curative treatment of metastatic ccRCC remains an unmet medical need [29–31]. A better understanding of molecular mechanisms that drive ccRCC cell migration and invasion is urgently needed. In this study, we presented evidence that the lncRNA MILIP is commonly upregulated in metastatic ccRCCs and its high expression is associated with poor outcome of ccRCC patients. Mechanistic investigations showed that MILIP forms an RNA-RNA duplex with the Snai1 mRNA that recruits YBX1 to facilitate translational activation of Snai1, thus promoting ccRCC cell migration and invasion through activation of the EMT. Moreover, we demonstrated that upregulation of MILIP in ccRCC cells is largely due to TFAP2C-mediated transcriptional activation.

Although MILIP exerts its biological effect on other types of cancer cells through binding to and repressing the tumor suppressor p53, the physical association between MILIP and p53 was not detected in ccRCC cells, consistent with the notion that lncRNAs function in a highly tissue- and cell type-dependent manner [19, 27]. MILIP-mediated regulation of ccRCC cell metastasis was associated with the EMT as shown by downregulation of the EMT hallmark pathway gene signature in ccRCC cells with MILIP knockdown. This was confirmed by upregulation of the epithelial marker E-cadherin and downregulation of multiple mesenchymal makers including N-cadherin and Vimentin caused by knockdown of MILIP [11]. In particular, MILIP promoted the expression of Snai1, a transcription factor key to activation of a variety of EMT genes that has been well-demonstrated to play an important role in ccRCC metastasis [32]. Of note, although Snai1 was downregulated in ccRCC cells at the protein level when MILIP was knocked down, its mRNA expression remained unaltered in ccRCC cells with or without knockdown of MILIP. Moreover, MILIP knockdown did not impinge on the turnover rate of the Snai1 protein, suggesting that MILIP plays a role in promoting Snai1 mRNA translation.

A number of lncRNAs have been shown to form RNA-RNA duplexes and thus regulate gene expression [20, 24]. In particular, several lnRNAs have been reported to be involved in regulation of mRNA translation [33, 34]. This is commonly accomplished through targeting specific translation factors such as eukaryotic translation initiation factor 4F (eIF4F) and eukaryotic translation initiation factor 4E (eIF4E) [33, 34]. For example, the lncRNA growth arrest specific 5 (GAS5) interacts with the translation initiation complex through binding to eIF4E, whereas the lncRNAs small nucleolar RNA host gene 1 (SNHG1) and SNHG4 similarly bind to eIF4E and dysregulate its function [33, 34]. Our results showed that the formation of an RNA-RNA duplex was necessary for MILIP to promote Snai1 mRNA translation, acting through the DFO within MILIP and the complementary MILIP-BR on the Snai1 mRNA, further highlighting the importance of RNA-RNA interactions in the lncRNA-mediated regulation of biological processes.

As a multifaceted protein, YBX1 acts typically as a transcription factor when located to the nucleus [28]. Nevertheless, it also exerts other biological functions in the cytoplasm [28]. In particular, cytoplasmic YBX1 differentially regulates the production of proteins associated with cell proliferation and the EMT process [13]. On the one hand, YBX1 competes with the eIF4E initiation complex for binding the 5' terminus of mRNAs involved in cell proliferation and thus inhibits their cap-dependent translation [13]. On the other hand, it interacts with the 5' UTRs of mRNAs encoding proteins associated with the EMT, such as the Snai1 and Slug, and thus promotes their cap-independent translation [13]. Our results showing that MILIP facilitates the binding of YBX1 to the Snai1 mRNA have provided further mechanistic insights into how the function of YBX1 in ccRCC cells is regulated. Of note, while YBX1 promotes cancer cell proliferation and survival in many cancer types, it functions primarily to enhance metastasis in ccRCC, consistent its role in translational activation of Snai1 in ccRCC cells [35–39]. Nevertheless, whether MILIP plays a similar role in regulating the effects of YBX1 on the expression of other EMT-associated proteins remains to be studied [13].

Although the proto-oncoprotein c-Myc transcriptionally activates MILIP in many types of cancer cells, it did not regulate MILIP expression in ccRCC cells, suggesting that MILIP is regulated through a different transcriptional mechanism [19]. Indeed, we found that MILIP expression in ccRCC cells is primarily activated by the transcription factor TFAP2C, an AP-2 transcription factor family member [40]. TFAP2C is upregulated in various cancer types and plays a role in tumorigenesis [41, 42]. In particular, it has been implicated in regulation of cancer cell metastasis [43–45]. Our results suggest that transcriptional activation of MILIP is involved in the TFAP2C-mediated regulation of metastasis of ccRCC.

A caveat of this study is that we were unable to apply our findings to spontaneous metastatic mouse models of ccRCC that would functionally substantiate the role of MILIP-mediated mechanism in ccRCC metastasis in vivo. This is largely due to the lack of similarities between human MILIP and transcripts of Mus musculus that precludes this approach. Similarly, whether MILIP forms RNA-RNA duplexes with other mRNAs remains an open question. Regardless, the results presented here demonstrate a signaling pathway encompassing TFAP2C, MILIP, YBX1 and Snai1 that promotes ccRCC metastasis and propose that MILIP may constitute a molecular target for treatment of late-stage ccRCC (Supplementary Fig. 6). The practical potentials of MILIP are supported by the findings in clinical samples, where MILIP expression is upregulated in metastatic ccRCC tissues and is associated with poorer prognosis of ccRCC patients.

## Conclusions

The TFAP2C-responsive lncRNA MILIP promotes ccRCC metastasis through forming an RNA-RNA duplex with the Snai1 mRNA to facilitate YBX1-mediated translational activation of Snai1. MILIP is upregulated in metastatic ccRCCs and high MILIP expression is associated with poor prognosis of ccRCC patients. These findings unveil a lncRNA-mediated mechanism contributing to ccRCC metastasis and implicate that targeting MILIP represents a novel avenue for the treatment of late-stage ccRCC.

## Supplementary Information

**Additional file 1**.

**Additional file 2: Supplementary Figure 1. Supplementary Figure 2. Supplementary Figure 3. Supplementary Figure 4. Supplementary Figure 5. Supplementary Figure 6.**

**Additional file 3: Supplementary Table 1.** List of antibodies.** Supplementary Table 2.** siRNA and shRNA sequences.** Supplementary Table 3.** List of qRT-PCR primer.** Supplementary Table 4.** List of RT-PCR primers and RNA pulldown probes.** Supplementary Table 5.** List of PCR primers for in vitro transcription.** Supplementary Table 6.** Summary of clinicopathological characteristics of the cohort of 14 clear cell renal cell carcinoma patients.** Supplementary Table 7.** Summary of proteins that interact with MILIP in Caki-1 cells detected using mass spectrometry.** Supplementary Table 8.** Summary of proteins that interact with MILIP in ACHN cells detected using mass spectrometry.

## Data Availability

The datasets supporting the conclusions of this article are available in the NCBI Sequence Read Archive (SRA) repository, [SRX14297667, SRX14297668, SRX14297669, SRX14297670, SRX14297671 and SRX14297672; https://www.ncbi.nlm.nih.gov/sra], the ProteomeXchange Consortium via the iProX partner repository, [PXD031980; http://proteomecentral.proteomexchange.org/cgi/GetDataset], the Gene Expression Omnibus (GEO) repository, [GSE22541; https://www.ncbi.nlm.nih.gov/geo/], and the Cancer Cell Line Encyclopedia (CCLE) [https://sites.broadinstitute.org/ccle/datasets]**.**

## Abbreviations

ccRCC: Clear cell renal cell carcinoma
lncRNA: Long non-coding RNA
TFAP2C or AP-2γ: Activating enhancer binding protein 2 (AP-2) gamma
EMT: Epithelial-to-mesenchymal transition
Snai1: Snail family transcriptional repressor 1
YBX1: Y-box binding protein 1
MILIP: C-Myc-inducible long noncoding RNA inactivating p53
MAFG: V-maf avian musculoaponeurotic fibrosarcoma oncogene homolog G
MAFG-AS1: MAFG antisense RNA 1
PLANE: Pan-cancer lncRNA activating NCOR2 responsive to E2F1
MELTF-AS1 or MFI2-AS1: Melanotransferrin antisense RNA 1
URRCC: Up-regulation in clear cell renal cell carcinoma
EGFL7: Epidermal growth factor-like protein 7
DMDRMR: DNA methylation–deregulated and RNA m6A reader–cooperating
IGF2BP3: Insulin-like growth factor 2 mRNA-binding protein 3
FBS: Fetal bovine serum
FFPE: Formalin-fixed paraffin-embedded
siRNAs: Small interfering RNAs
shRNA: Short hairpin RNA
BSA: Bovine serum albumin
RT: Room temperature
RIP: RNA immunoprecipitation
dChIRP: Domain-specific chromatin isolation by RNA purification
ISH: In situ hybridization
DAB: 3,3’-Diaminobenzidine
RS: Reactive score
ChIP: Chromatin immunoprecipitation assay
GEO: Gene Expression Omnibus
DFS: Disease-free survival
CCLE: Cancer Cell Line Encyclopedia
ChIP-seq: Chromatin immunoprecipitation sequencing
ENCODE: Encyclopedia of DNA Elements
TFAP2C-BR: TFAP2C-binding region
GSEA: Gene set enrichment analysis
RNA-seq: RNA-sequencing
MMP2: Matrix metalloproteinase-2
Dox: Doxycycline
E2: Exon 2
CSD: Cold shock domain
CHX: Cycloheximide
MILIP-BR: MILIP-binding region
5’UTR: 5′Untranslated region
DFO: Duplex-forming oligonucleotides
MILIP-ΔDFO: MILIP mutant lacking the DFO
eIF4F: Eukaryotic translation initiation factor 4F
eIF4E: Eukaryotic translation initiation factor 4E
GAS5: Growth arrest specific 5
SNHG1: Small nucleolar RNA host gene 1
