# Supplementary material for: LncRNA MILIP links YBX1 to translational activation of Snai1 and promotes metastasis in clear cell renal cell carcinoma
Source: J Exp Clin Cancer Res. 2022 Aug 26;41:260. doi: 10.1186/s13046-022-02452-9 (PMC9414127; doi:10.1186/s13046-022-02452-9)

Supplementary Figure 1

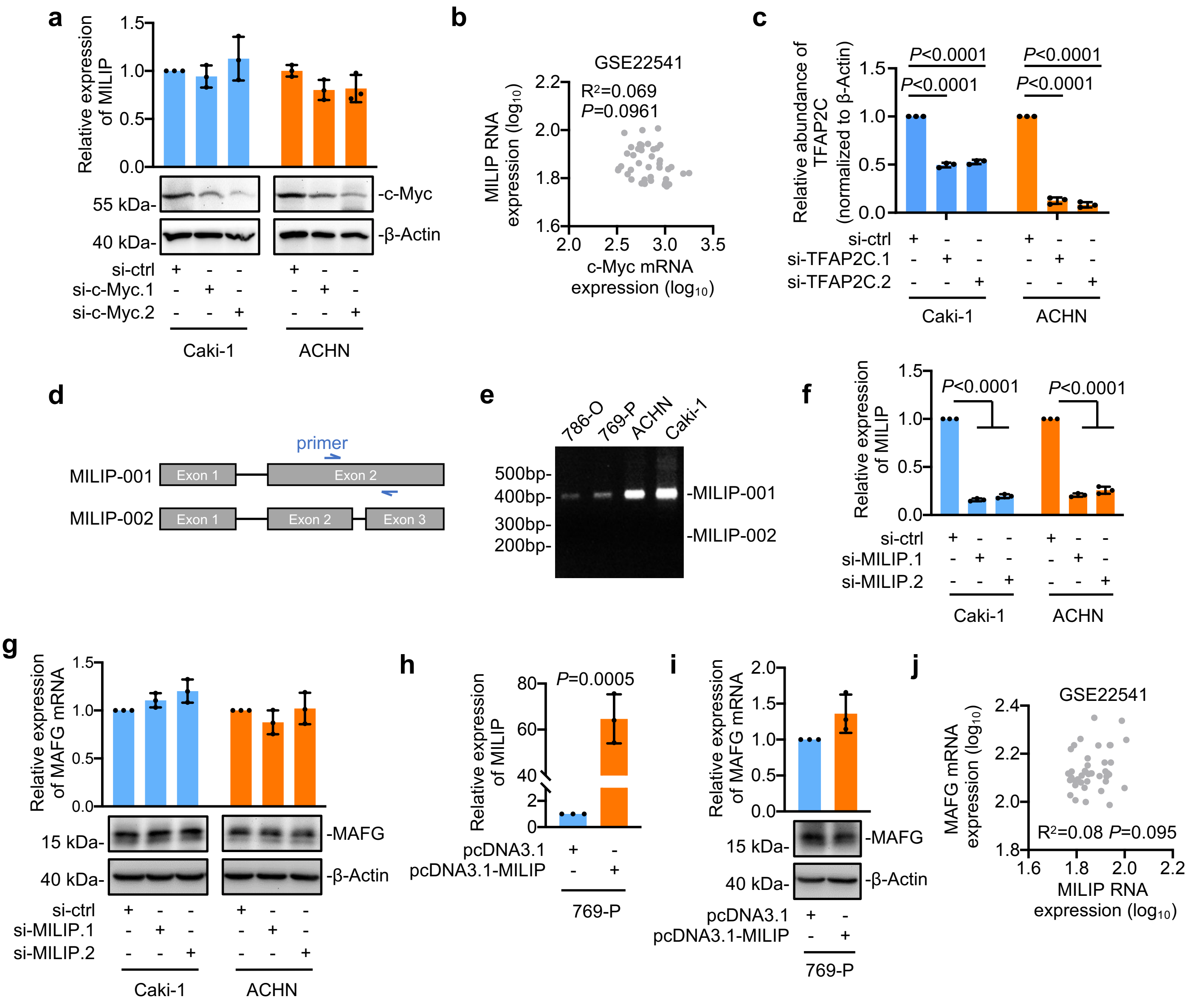

Supplementary Figure 2

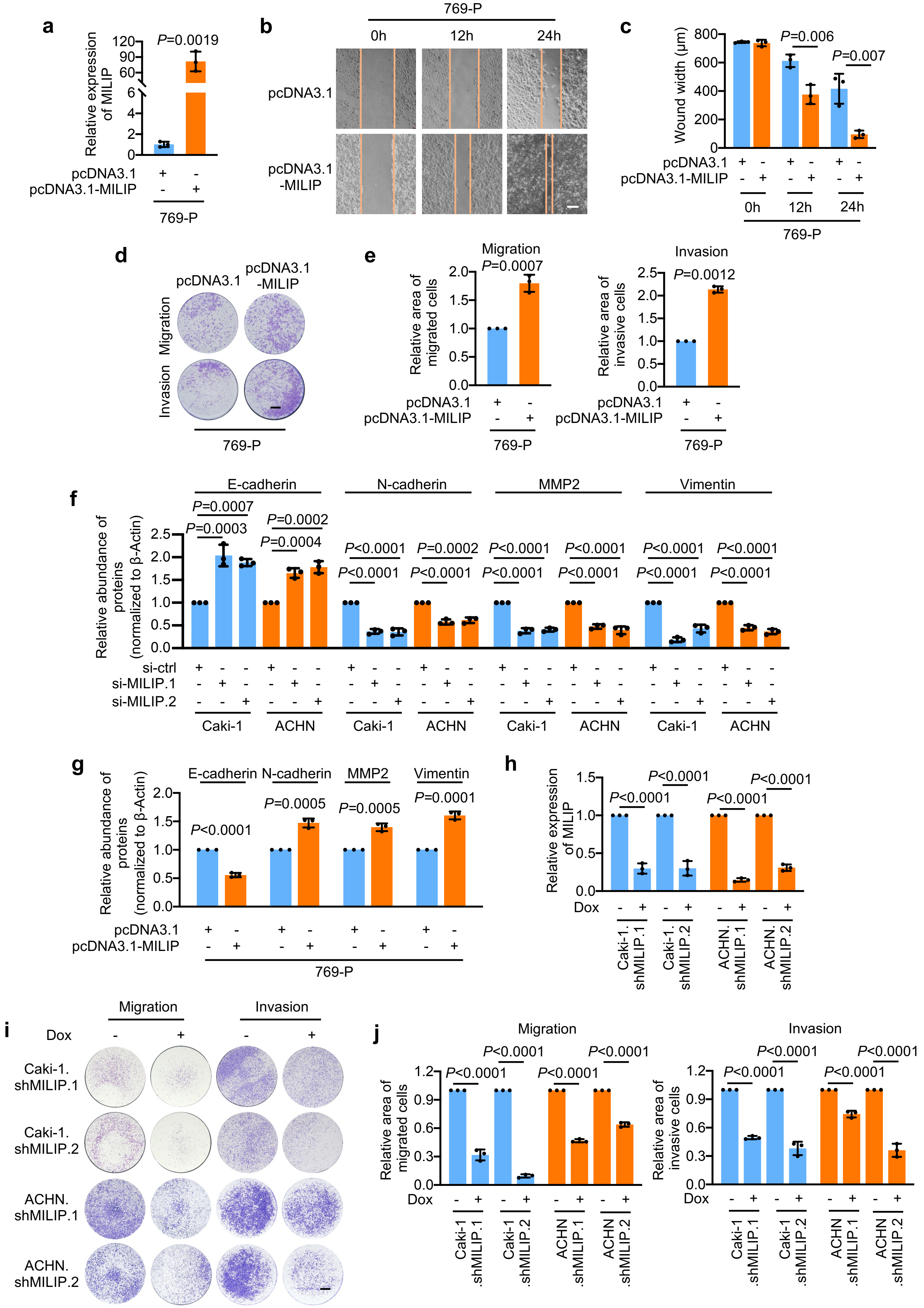

Supplementary Figure 3

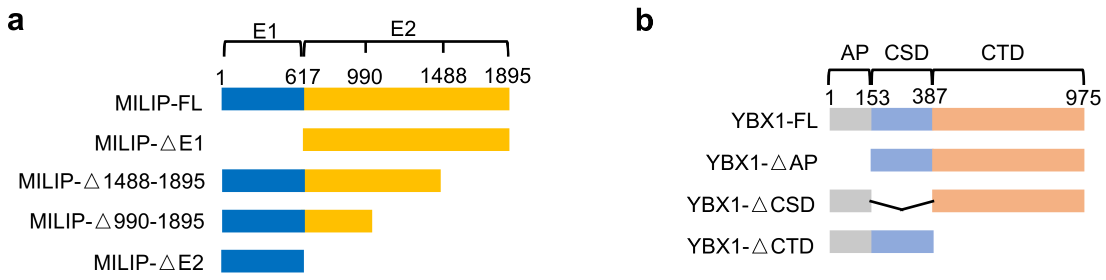

Supplementary Figure 4

a

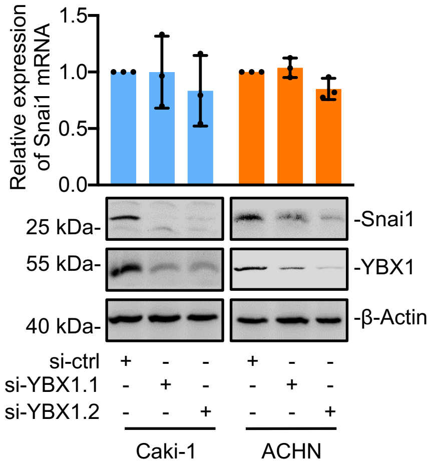

Supplementary Figure 5

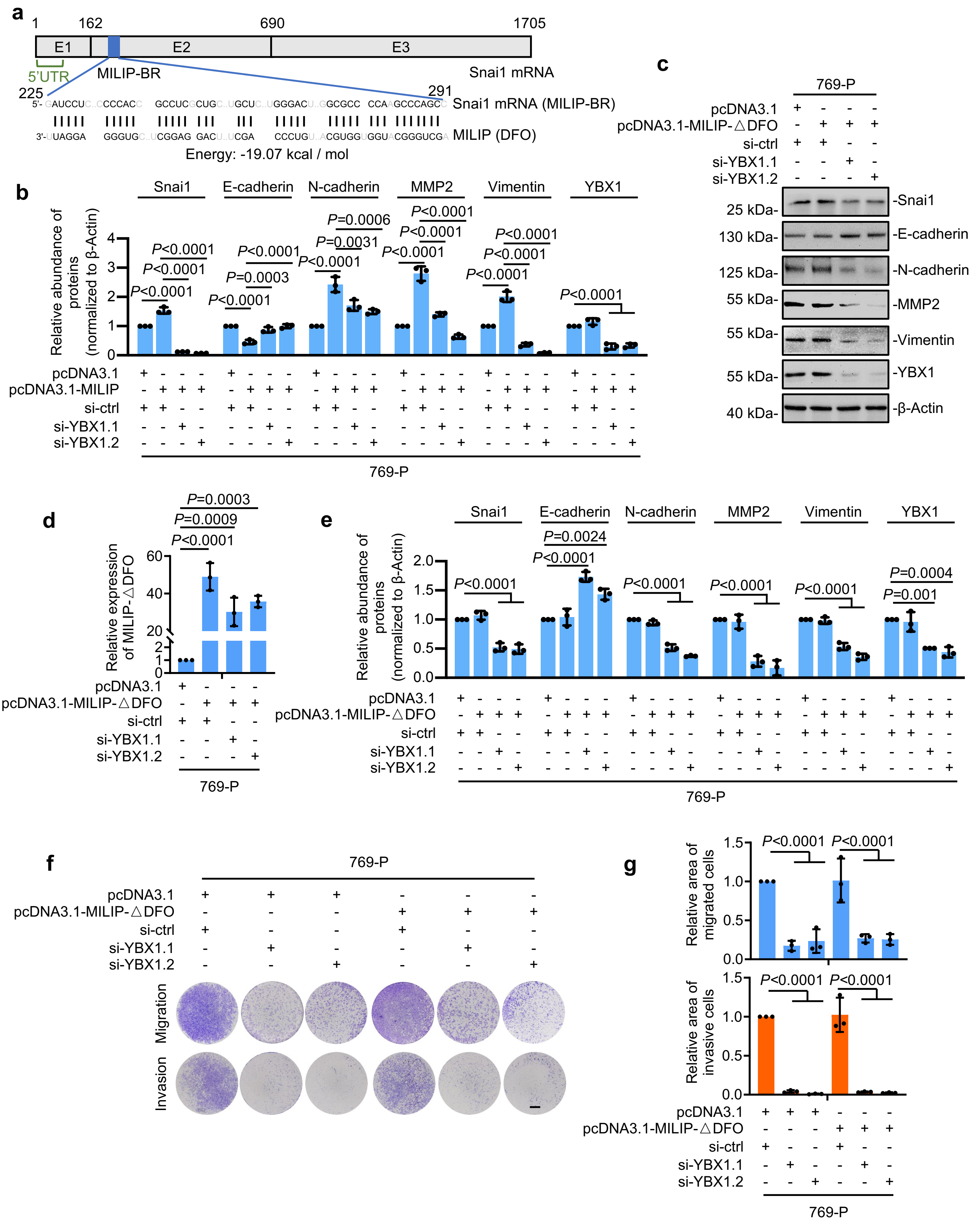

Supplementary Figure 6

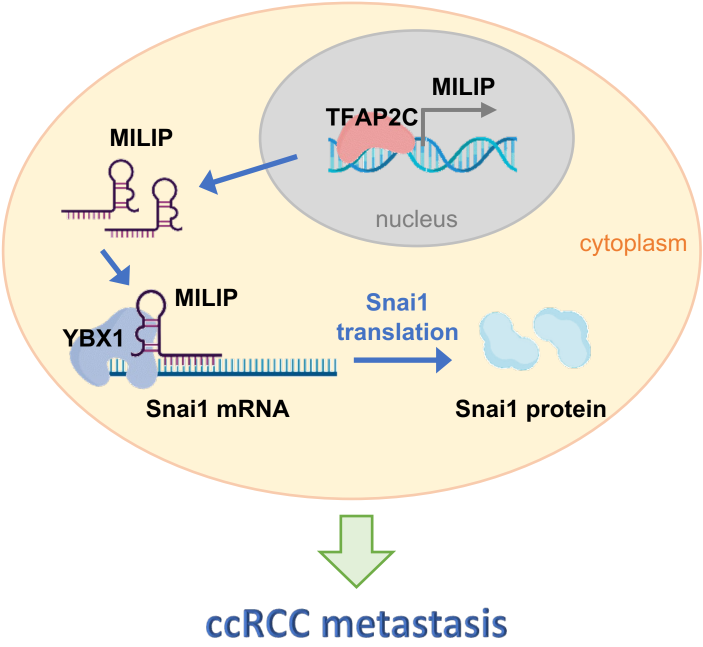

Supplement: Supplementary file 2 — Additional file 2: Supplementary Figure 1. Supplementary Figure 2. Supplementary Figure 3. Supplementary Figure 4. Supplementary Figure 5. Supplementary Figure 6. [file 13046_2022_2452_MOESM2_ESM.pdf]
