# Supplementary material for: LncRNA MILIP links YBX1 to translational activation of Snai1 and promotes metastasis in clear cell renal cell carcinoma
Source: J Exp Clin Cancer Res. 2022 Aug 26;41:260. doi: 10.1186/s13046-022-02452-9 (PMC9414127; doi:10.1186/s13046-022-02452-9)
Supplement: Supplementary file 3 — Additional file 3: Supplementary Table 1. List of antibodies. Supplementary Table 2. siRNA and shRNA sequences. Supplementary Table 3. List of qRT-PCR primer. Supplementary Table 4. List of RT-PCR primers and RNA pulldown probes. Supplementary Table 5. List of PCR primers for in vitro transcription. Supplementary Table 6. Summary of clinicopathological characteristics of the cohort of 14 clear cell renal cell carcinoma patients. Supplementary Table 7. Summary of proteins that interact with MILIP in Caki-1 cells detected using mass spectrometry. Supplementary Table 8. Summary of proteins that interact with MILIP in ACHN cells detected using mass spectrometry. [file 13046_2022_2452_MOESM3_ESM.pdf]

Supplementary Tables

Supplementary Table 1. List of antibodies

**Antibody (Ab)**	**Catalogue No.**	**Company**	**Dilution**
E-cadherin Mouse mAb[1]	14472S	Cell Signaling Technology	1:1000
N-cadherin Rabbit pAb[2]	AF0243	Beyotime	1:1000
MMP2 Rabbit pAb	AF0234	Beyotime	1:1000
Vimentin Rabbit mAb	AF1975	Beyotime	1:2000
Actin Rabbit pAb	20536-1-AP	Proteintech	1:1000
YBX1 Rabbit mAb	ab76149	Abcam	1:1000
p53 Rabbit pAb	10442-1-AP	Proteintech	1:1000
Lamin A/C Rabbit pAb	AF7350	Beyotime	1:1000
GAPDH Mouse mAb	AF5009	Beyotime	1:1000
Snai1 Rabbit pAb	3879S	Cell Signaling Technology	1:1000
c-Myc Rabbit pAb	9402	Cell Signaling Technology	1:1000
TFAP2C Rabbit pAb	ab218107	Abcam	1:1000
Normal rabbit IgG	A7016	Beyotime	1:1000
MAFG Rabbit pAb	GTX114541	GeneTex	1:1000
GFP Mouse mAb	66002	Proteintech	1:1000
Goat Anti-Mouse IgG (H+L)-HRP Conjugate	A0216	Beyotime	1:1000
Goat Anti-Rabbit IgG (H+L)-HRP Conjugate	A0208	Beyotime	1:1000

[1]mAb: monoclonal antibody
[2]pAb: polyclonal antibody

Supplementary Table 2. siRNA and shRNA sequences

**MILIP**	siRNA1/shRNA1 GGTAACATAGAGACCCTAT
	siRNA2/shRNA2 GGAGTCAGGGCAATTCCAA
**YBX1**	siRNA1 GGACGGCAATGAAGAAGAT
	siRNA2 CCACGCAATTACCAGCAAA
**c-Myc**	siRNA1 CCTGAGACAGATCAGCAACAAC
	siRNA2 GGACTATCCTGCTGCCAAG
**Snai1**	siRNA1 TGTAGTTAGGCTTCCGATTGG
	siRNA2 TACTTCTTGACATCTGAGTGG
**TFAP2C**	siRNA1 TATTTAGACGTAGAGCTGAGG
	siRNA2 TTCTTTACACAGTTGCTGGGC

Supplementary Table 3. List of qRT-PCR primers

**MILIP**	Forward: AGAACCGCGAAAGGCTACTG
	Reverse: CACTTAAAGCCGGTCGTGGA
**Neat1**	Forward: GCATACGCAGCAGATCAGCAT
	Reverse: CCCACAATATAGGCATTTACAAGG
**GAPDH**	Forward: AGCCACATCGCTCAGACAC
	Reverse: GCCCAATACGACCAAATCC
**MAFG**	Forward: AGGAGATCGTCCAGCTGAAGCA
	Reverse: TCTGCTTCTCCAGCTCCTCCTT
**β-Actin**	Forward: CTCTTCCAGCCTTCCTTCCT
	Reverse: AGCACTGTGTTGGCGTACAG
**PLANE**	Forward: TGAGACGACATCCCTTCCAG
	Reverse: GGCCCTGAAATGACTTGCTC
**Snai1**	Forward: TGCCCTCAAGATGCACATCCGA
	Reverse: GGGACAGGAGAAGGGCTTCTC

Supplementary Table 4. List of RT-PCR primers and RNA pulldown probes

**MILIP isoforms**	Forward: TCTCCACGACCGGCTTTAAGTG
	Reverse: GAACCGATGGGGTCTTAAGGTCAG
**ChIP-TFAP2C-BR**	Forward: CGGCCTTTTAGTCCCCGAGGC
	Reverse: GAGGAAAGGGTGGGGTCGAGC
**MILIP-E1**	Forward: GAGACTTCGCTGCTTGTTGG
	Reverse: GCTGGCGGGGCGGCCACCCTTC
**MILIP-E2**	Forward: ACTTCGCTGCTTGTTGGAGA
	Reverse: GGAGAAAGTCCAGACACGGG
**MILIP-biotin-S-probes**	Scramble 1: GACAATACTCGACAGGCTCC
	Scramble 2: GAGCGAAGGTTATGTCGACC
	Scramble 3: GTGAACAACGCGAGTTGTGGA
**MILIP-biotin-AS-probes**	Antisense 1: TGACCACGGAACACCTTCAG
	Antisense 2: ACTGTGTCCAGGGACAAGTG
	Antisense 3: TGAGAGGGATGCTTGGAAACC
**Snai1-biotin-S-probes**	Scramble 1: CAGGACATACAGATCCCACTA
	Scramble 2: ACTCCACCATGGAATATGAGA
**Snai1-biotin-AS-probes**	Antisense 1: TGTAGTTAGGCTTCCGATTGG
	Antisense 2: TACTTCTTGACATCTGAGTGG

Supplementary Table 5. List of PCR primers for *in vitro* transcription

**MILIP**	Forward: AATACGACTCACTATAGGGATGCGCAACCCGCGCGCCGT
	Reverse: TCCCCAGCAGGCCAGGTGGGC
**MILIP-ΔE1**	Forward: AATACGACTCACTATAGGGAGAGAGCTGAAGGTGTTCCGT
	Reverse: TCCCCAGCAGGCCAGGTGGGC
**MILIP-Δ1488-1895**	Forward: AATACGACTCACTATAGGGATGCGCAACCCGCGCGCCGT
	Reverse: CTTGCCAGCTGGGGCCCTTGC
**MILIP-Δ990-1895**	Forward: AATACGACTCACTATAGGGATGCGCAACCCGCGCGCCGT
	Reverse: GATGCCCGGGAGGTGCCTG
**MILIP-ΔE2**	Forward: AATACGACTCACTATAGGGATGCGCAACCCGCGCGCCGT
	Reverse: CTGCTGGCGGGGCGGCCACCCTTC

Supplementary Table 6. Summary of clinicopathological characteristics of the cohort of 14 clear cell renal cell carcinoma patients

Characteristics	Cases	MILIP abundance in clear cell renal cell carcinoma (RS[1])	*P* value[2]
**Gender**	14		
Male	11	0.46±0.23[(3)]	0.3492
Female	3	0.99±0.47	
**Age**	14		
≥59[(4)]	7	0.6±0.36	0.8877
<59	7	0.54±0.24	
**TNM**	14		
I-III	10	0.16±0.11	0.0002
IV	4	1.58±0.32	
**Distant metastasis**	14		
M0	9	0.05±0.03	<0.0001
M1	5	1.50±0.26	

[1]RS: Reactive score

[2]Two-tailed Student's *t*-test; a *P* value less than 0.05 was considered statistically significant

[3]Data shown are mean ± s.e.m

[4]The median age of the patients in this cohort was 59

Supplementary Table 7. Summary of proteins that interact with MILIP in Caki-1 cells detected using mass spectrometry

No.	Entry name	Coverage (%)	MW (kDa)	Score
1	YBX1	49	35.9	77.94
2	KRT1	27	66	47.62
3	KRT2	20	65.4	32.39
4	KRT10	16	58.8	32.13
5	YBX3	14	40.1	27.04
6	KRT5	12	62.3	19.5
7	KRT6B	10	60	18.84
8	KRT9	15	62	16.77
9	RPA1	12	68.1	15.42
10	KRT14	10	51.5	13.91
11	RBMS1	8	44.5	9.61
12	XRCC1	7	69.4	6.86
13	KRT77	5	61.9	6.82
14	RBMS2	6	43.9	3.62
15	RPL4	3	47.7	2.76
16	RBM14	2	69.4	2.15
17	HSPD1	2	61	1.63
18	RBFOX2	2	41.3	0
19	RBMS3	2	47.8	0

Supplementary Table 8. Summary of proteins that interact with MILIP in ACHN cells detected using mass spectrometry

No.	Entry name	Coverage (%)	MW (kDa)	Score
1	YBX1	54	35.9	247.05
2	YBX3	36	40.1	105.54
3	KRT1	37	66	79.48
4	KRT2	36	65.4	61.06
5	KRT10	25	58.8	45.41
6	KRT9	17	62	23.54
7	KRT5	13	62.3	22.30
8	KRT14	20	51.5	19.13
9	RBMS1	13	44.5	17.29
10	ALB	4	69.3	6.73
11	IGF2BP3	7	63.7	5.95
12	ETFA	6	35.1	5.58
13	ATP5F1A	2	59.7	4.73
14	RBFOX1	3	42.8	4.10
15	RNF146	7	38.9	3.81
16	ACTB	5	41.7	2.29
17	RBMS2	3	43.9	1.87
